# Urinary melatonin-sulfate/cortisol ratio and the presence of prostate cancer: A case-control study

**DOI:** 10.1038/srep29606

**Published:** 2016-07-08

**Authors:** Shu-Yu Tai, Shu-Pin Huang, Bo-Ying Bao, Ming-Tsang Wu

**Affiliations:** 1 Graduate Institute of Medicine, College of Medicine, Kaohsiung Medical University, Kaohsiung, Taiwan; 2 Department of Family Medicine, School of Medicine, College of Medicine, Kaohsiung Medical University, Kaohsiung, Taiwan; 3 Department of Family Medicine, Kaohsiung Municipal Ta-Tung Hospital, Kaohsiung, Taiwan; 4 Department of Family Medicine, Kaohsiung Medical University Hospital, Kaohsiung Medical University, Kaohsiung, Taiwan; 5 Research Center for Environmental Medicine, Kaohsiung Medical University, Kaohsiung, Taiwan; 6 Department of Urology, Kaohsiung Medical University Hospital, Kaohsiung Medical University, Kaohsiung, Taiwan; 7 Department of Urology, Faculty of Medicine, College of Medicine, Kaohsiung Medical University, Kaohsiung, Taiwan; 8 Institute of Biomedical Science, National Sun Yat-Sen University, Kaohsiung, Taiwan; 9 Department of Pharmacy, China Medical University, Taichung, Taiwan; 10 Sex Hormone Research Center, China Medical University Hospital, Taichung, Taiwan; 11 Department of Nursing, Asia University, Taichung, Taiwan; 12 Department of Public Health, Kaohsiung Medical University, Kaohsiung Medical University, Kaohsiung, Taiwan; 13 Center of Environmental and Occupational Medicine, Kaohsiung Municipal Hsiao-Kang Hospital, Kaohsiung Medical University, Kaohsiung, Taiwan

## Abstract

The circadian-related hormones, melatonin and cortisol, have oncostatic and immunosuppressive properties. This study examined the relationship between these two biomarkers and the presence of prostate cancer. We measured their major metabolites in urine collected from 120 newly diagnosed prostate cancer patients and 240 age-matched controls from January 2011 to April 2014. Compared with patients with lower urinary melatonin-sulfate or melatonin-sulfate/cortisol (MT/C) ratio levels, those with above-median levels were significantly less likely to have prostate cancer (adjusted OR (aOR) = 0.59, 95% CI = 0.35–0.99; aOR = 0.46, 95% CI: 0.27–0.77) or advanced stage prostate cancer (aOR = 0.49, 95% CI = 0.26–0.89; aOR = 0.33, 95% CI = 0.17–0.62). The combined effect of both low MT/C ratios and PSA levels exceeding 10 ng/ml was an 8.82-fold greater likelihood of prostate cancer and a 32.06-fold greater likelihood of advanced stage prostate cancer, compared to those with both high MT/C ratios and PSA levels less than 10 ng/ml. In conclusion, patients with high melatonin-sulfate levels or a high MT/C ratio were less likely to have prostate cancer or advanced stage prostate. Besides, a finding of a low MT/C ratio combined with a PSA level exceeding 10 ng/ml showed the greatest potential in detecting prostate cancer and advanced stage prostate cancer.

Twenty-four hour biological rhythms allow organisms to tune their physiology to the daily cycle of sunlight and darkness. It has been suggested that the disruption of biological rhythms, or the mismatch between the sleep/wake cycle and the endogenous circadian timing system, may contribute to short- and long-term adverse effects as well as cancer risk^1^. Melatonin, a circadian and circannual time signal secreted by the human pineal gland^2^,^3^, is known to have significant anticancer potential and has been found to have chemopreventive, oncostatic, and tumor inhibitory effects in a variety of *in vitro* and *in vivo* experimental models of neoplasia^4^,^5^,^6^. It is also an anti-physical stress hormone^7^,^8^, an immunomodulatory agent^9^,^10^, and a powerful and effective endogenous hydroxyl radical scavenger^11^,^12^, and, as such, it has both direct and indirect anticancer effects.

Most cancer research on melatonin in the past three decades has largely centered on breast cancer^13^,^14^,^15^,^16^,^17^,^18^,^19^, the most common female cancer^20^. However, there are few studies investigating the potential role of melatonin in human prostate cancer, the most commonly diagnosed non-cutaneous cancer in men^20^. Prostate and breast cancer are similar in that both are sex hormone-dependent^21^. Many studies have found an association between circadian disruption, including melatonin levels, and breast cancer, but few studies have investigated the association between circadian disruption or sleep loss and prostate cancer risk, which has been found to be positive^22^.

The fact that patients with prostate cancer have lower melatonin levels than patients with BPH suggests that melatonin may protect against disease severity^23^. Melatonin has been found to have potential in the chemoprevention and treatment of prostate cancer *in vitro* studies^24^. Cortisol is another hormone important to circadian regulation^25^,^26^. One previous study revealed that cortisol secretion patterns may be impacted by shift work, which is known to cause circadian disruption^27^. This hormone has been found to influence cancer risk through its effects on immune function^28^,^29^. It is thus possible that carcinogenicity of circadian disruption can broadly affect many sites among both men and women via melatonin and cortisol levels. Therefore, we conducted this case-control study to examine the relationship between two urine biomarkers of circadian regulation hormones, melatonin and cortisol level, and the presence of prostate cancer and their impacts on the clinical stages of prostate cancer. Because PSA is also associated with the presence or relapse of prostate cancer, this antigen was also included to examine the interaction effect of circadian regulation hormones and PSA on prostate cancer.

## Results

In total, we recruited 137 pathology-proved prostate cancer patients and 274 male controls (average: 70.53 years; range 45 to 85 years) between January 2011 and April 2014. After excluding cases with other cancers or cases without available individual matches, we were left with 120 cases and 240 controls to include in our final analysis. As can be seen in Table 1, summary of participant characteristics, the only significant difference between the cases and controls were PSA levels (*p* < 0.001). Most (75.0%) of those included in the study were 65 years old or older, had high school or college educations or less (76.7%), were married (87.7%), had no family history of prostate cancer (85.5%), did not smoke (70.1%), use alcohol (88.8%), chew betel nut (98.3%), or use vitamin D supplements (96.6%). There were no significant differences between the two groups with regard to these characteristics.

**Figure 1:**
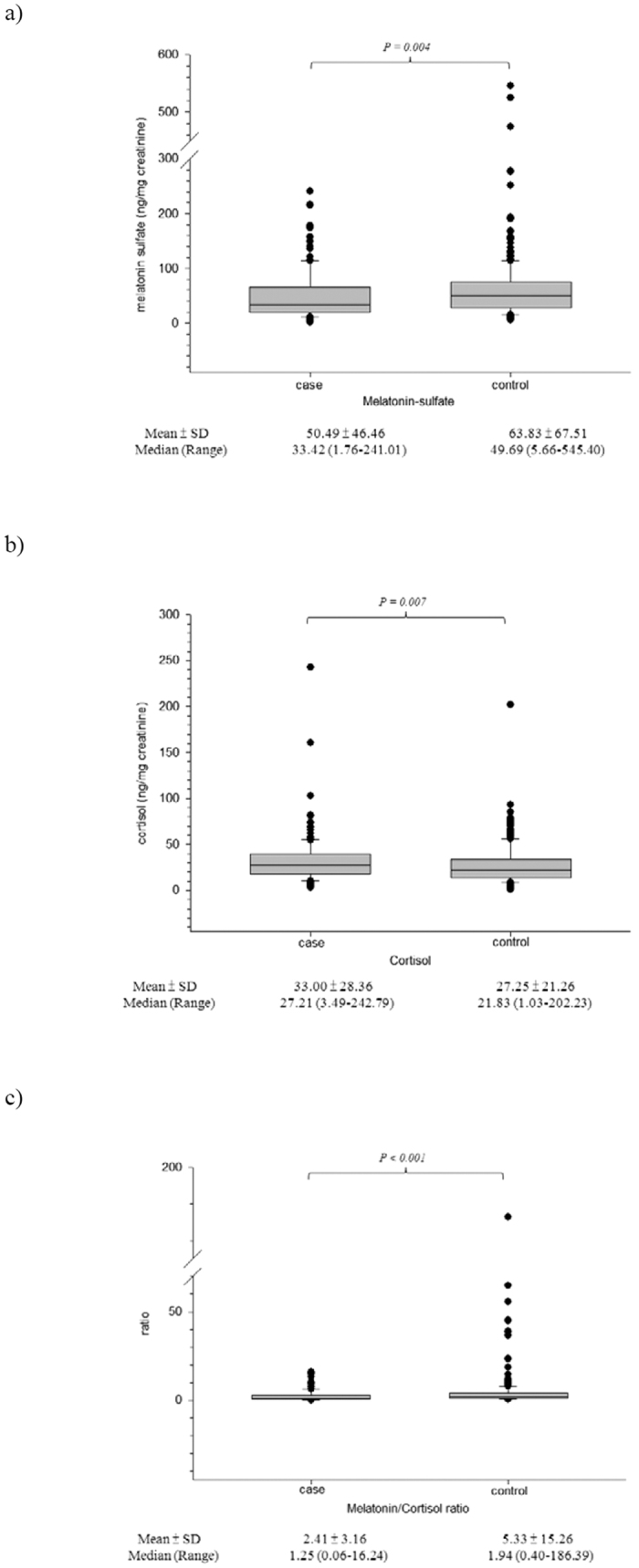
Urinary biomarkers of circadian hormone between case and control groups. (**a**) melatonin; (**b**) cortisol; (**c**) ratio of melatonin/cortisol.

Figure 1 shows the differences in the urinary biomarkers between cases and controls. Compared to the controls, the cases had a significantly lower levels of the urinary melatonin-sulfate (mean ± SD) (50.49 ± 46.46 *vs.* 63.83 ± 67.51 ng/mg creatinine, *p* = 0.004) and lower MT/C ratios (2.41 ± 3.16 *vs.* 5.33 ± 15.26, *p* < 0.001). In contrast, the cases had significantly higher mean urinary cortisol levels than the controls (33.00 ± 28.36 *vs.* 27.25 ± 21.26 ng/mg creatinine, *p* = 0.007).

After adjusting for other covariates, we found that subjects with high urinary melatonin-sulfate or MT/C ratios were significantly less likely to have prostate cancer compared to those with low urinary melatonin-sulfate or MT/C ratios (adjusted OR (aOR) = 0.59, 95% CI = 0.35–0.99 and aOR = 0.46, 95% CI = 0.27–0.77, respectively) (Table 2). In addition, subjects with both high MT/C ratio and a pre-operative PSA level exceeding 10 ng/ml were 3.61 fold more likely (95% CI = 1.62–8.07) to have prostate cancer, when compared to those with both high MT/C ratio and a pre-operative PSA level less than 10 ng/ml. (Table 2). The risk was even higher in the group with low MT/C ratios and pre-operative PSA levels exceeding 10 ng/ml (aOR = 8.82), (95% CI = 3.98–19.55). A similar risk pattern was also observed among the subjects when we combined risk of melatonin sulfate or cortisol and PSA level (Supplementary Table S1).

Categorizing prostate cancer by clinical stage (localized and advanced), we found significant differences of urinary biomarkers when comparing advanced cancer and control groups. No multiplicative scale of interaction was obtained for the MT/C ratio and PSA level (Table 3). In addition, we found evidence of a combined effect of low MT/C ratio and the pre-operative PSA levels exceeding 10 ng/ml in the advanced cancer group compared with either control group or localized cancer group (Table 3). A similar trend pattern was also observed among the subjects when combining melatonin sulfate or cortisol and PSA level (Supplementary Table S2).

## Discussion

In this case-control study, we found an inverse relation between first morning urinary melatonin-sulfate levels and the melatonin/cortisol (MT/C) ratio and the presence of prostate cancer overall and advanced stage prostate cancer.

Most prior studies have focused on evaluating the association between melatonin levels and breast cancer. Very few have focused on the association between melatonin and prostate cancer. One cross-sectional study by Bartsch *et al*. reported that men with prostate cancer had lower melatonin levels than men with BPH^23^. Another case-cohort study in an Icelandic population, conducted by Sigurdardottir *et al*., found that subjects with below-median morning pre-diagnostic 6-sulfatoxymelatonin (aMT6s) levels had a statistically significant 4-fold increased risk for advanced disease, compared to those with above-median levels (Hazard ratio = 4.04; 95% CI = 1.26–12.98)^30^. However, that study did not find a significant association between morning urinary aMT6s levels and prostate cancer risk overall. Our study contributes additional information about circadian hormones and the presence of prostate cancer in an Asian population.

The protective effect of melatonin on cancer risk may be related to its inhibition of cancer cell growth, its protection of cells from DNA damage, and its promotion of repair of DNA damage once it has occurred^1^,^31^,^32^. Recently, Blask *et al*. conducted a series of experiments using both steroid receptor-positive and -negative human breast cancer xenografts in rats and found an inverse relationship between melatonin level and tumor activity^33^,^34^. The same research group also reported similar results with prostate cancer xenografts^35^. Other studies have reported a reduction in growth of malignant prostate tumor cells achieved by the administration of both pharmacologic and physiologic doses of melatonin^24^,^36^,^37^,^38^, although their findings have not always been consistent^39^,^40^.

Our study also measured cortisol, another important circadian hormone secreted by the adrenal cortex. Cortisol has been found to help regulate both immunity and inflammation; a deficiency in this hormone may result in an unresponsive immune system and an overabundance of the hormone may suppress immune responses^41^. Moreover, chronic dysregulation of the circadian cortisol rhythm has been associated with higher levels of inflammation^28^, and such inflammation may play a critical role in carcinogenesis^42^. Mirick *et al*. found that circadian disruption can impact cortisol secretion patterns, which may in turn affect cancer risk^27^. Although this study did not find a significant association between one-spot morning urinary cortisol level and prostate cancer, we did find an inverse association between the MT/C ratio and the presence of prostate cancer and advanced stage prostate cancer. Previous studies have found MT/C ratio to be related to different types of depression and the severity of depression^43^,^44^. This is the first study to evaluate the association between the MT/C ratio and the presence of prostate cancer.

It is difficult to decide clinically whether to perform an invasive prostate biopsy for subjects with abnormal PSA levels. Our finding that the combination of low MT/C ratios and PAS levels in excess of 10 ng/ml conferred the highest tendency that a person may have prostate cancer and advanced-stage disease prostate cancer suggests that we might consider the biomarker of MT/C ratio in urine to be an additional tool for judging whether the prostate biopsy is needed or not, when PSA levels are elevated. Further study is needed to investigate the clinical utility of the combining PSA and MT/C to detect the presence of prostate cancer and stage the disease.

A high proportion of controls in this study had PSA levels exceeding10 ng/ml. Increased PSA concentrations are found in the sera of patients with BPH or patients with prostate cancer, respectively^45^. An estimated 50% of men have histologic evidence of BPH by age 50, 75% by age 80, and 90% of men by age 85 years^46^. About 99% of prostate cancer cases occur in those over the age of 50^47^. This is of particular concern in older men, where BPH is more prevalent, as BPH increases gland volume which in turn increases the PSA level as well as the number of sampling errors associated with prostate biopsy^48^,^49^. For men with PSA 4–10 ng/mL, the detection rate of PCa in Caucasian men may be as high as 40%^50^,^51^ but only 20% in Chinese men^52^. Although the inclusion criteria for our control group was either unremarkable digital rectal exams or remarkable digital rectal exams but histologically confirmed BPH, our control group may have included some men with undiagnosed prostate cancer. However, the inclusion of such patients as controls would only lead to underestimations of the true association.

The strengths of our study are that we were able to consider the most important prostate cancer risk factors, including clinical stage and PSA level in our analyses. Furthermore, we considered two important circadian biomarkers and found the M/C ratio is more relevant to prostate cancer. This study also has several limitations. One limitation is that it is a cross-sectional case-control study, so no clear causal relationship can be inferred. There is a likelihood of a reverse causality between circadian hormones and the presence of prostate cancer, if circadian disruption and/or sleep disruption following a cancer diagnosis results in the decline of melatonin levels. Another limitation is that we used one single measurement of one-spot morning urinary biomarkers, which may not represent long-term exposure level. Although we had broad information on various covariates and were able to control for possible confounders, we still lacked information on factors such as have sleep disorders or medication use, etc. Finally, the exposures of interest were collected using a questionnaire, which may lead to some recall bias.

## Conclusion

Lower morning melatonin-sulfate levels or MT/C ratio were associated with the presence of prostate cancer. In addition, patients with both low MT/C ratios and PSA levels exceeding 10 ng/ml appeared to be far more likely to have prostate cancer and advanced stage disease. Because this is a cross-sectional case-control study, we could not establish a definite causal relationship between urinary MT/C ratio and risk of prostate cancer. Larger prospective cohort studies are needed to verify these findings.

## Methods

### Study populations

This hospital-based case-control study was conducted at Kaohsiung Medical University Hospital (KMUH), a medical center in Kaohsiung City, a harbor city located on the southwest coast of Taiwan. Cases were men with incident, histologically confirmed adenocarcinoma of prostate with no previous diagnosis of cancer in other sites between January 2011 and April 2014. Each case patient was age-matched (within 2 years) with two healthy men who came in for health check-ups at our Department of Preventive Medicine the same month that the case were recruited after verifying the absence of any neoplastic diseases. The controls had either unremarkable digital rectal exams or remarkable digital rectal exams but histologically confirmed benign prostatic hyperplasia (BPH).

### Data collection

#### Overview

Reference date for the cases was defined as date of pathology proof of prostate cancer. Controls were matched within the same month, the reference month. Subjects in both groups answered in-person interviews conducted by trained interviewers using standardized questionnaires collecting socio-demographic information (age, education, and marital status), family history of prostate cancer (PCa) and lifestyle (use of tobacco, alcohol, betel chewing, and vitamin D supplements). The medical charts of all cases were reviewed to collect data on serum prostate-specific antigen (PSA) level at diagnosis and disease stage. The medical charts of controls were reviewed to collect measured PSA results. All study procedures were reviewed and approved by the Institutional Review Board of the Kaohsiung Chung-Ho Memorial Hospital (KMUH-IRB-20110101). All the methods were carried out in accordance with the approved guidelines, and written informed consent was obtained from all subjects.

#### Urine sample collection

After their interviews, both case and control subjects provided the first-spot morning urine samples after waking up. The samples were divided into labeled cry-tubes of 4.5 mL volume each and then immediately stored at −20 °C. Urine specimens from case subjects and matched control subjects were handled identically and assayed on the same day and in the same run. All samples were taken out of the freezer simultaneously and sent to the laboratory in the same parcel on dry ice. Laboratory personnel were blinded to the case-control status of all specimens. Analytic error was controlled for by including two standard samples in each assay. The morning urinary measurements showed good sensitivity and specificity in identifying individual differences in nocturnal plasma melatonin levels^53^.

#### Assessment of urinary melatonin-sulfate

Urinary melatonin sulfate was measured by solid phase enzyme-linked immunosorbent assay (Melatonin Sulfate ELISA Kit, GenWay Biotech Inc., San Diego, USA). Assay sensitivity was 1.0 ng/dL, and the upper limits of intra- and inter-assay coefficients of variation ranged 5.2–12.2% and 5.1–14.9%, respectively, at 5.8–204 ng/dL and 12.4–220 ng/dL. Optical density was measured using an automatically photometer (ELx808™ Absorbance Microplate Reader) at 450 nm.

#### Assessment of urinary cortisol

Urine cortisol was measured by chemiluminescent immunoassay performed on an ADVIA Centaur XP Immunoassay System analyzer (Siemens Healthcare Diagnostics Ltd., Frimley, Camberley, UK). The ADVIA Centaur Cortisol assay is standardized using internal standards manufactured analytically traceable to gas chromatography-mass spectroscopy (GCMS). Assay sensitivity was 0.2 μg/dL. The upper limits of intra- and inter-assay coefficients of variation were 2.89–3.82% and 1.86–5.45%, respectively, at 3.88–37.15 ng/dL. Urinary creatinine levels were measured by the modified Jaffe reaction. Urinary melatonin-sulfate and cortisol levels were corrected by urinary creatinine levels^53^,^54^.

### Statistical analysis

Urinary melatonin-sulfate/cortisol (MT/C) ratio was calculated by urinary melatonin-sulfate levels divided by urinary cortisol levels as a basis for measuring the combined impact of melatonin and cortisol levels simultaneously on the prostate cancer. Wilcoxon rank-sum test was used to compare the differences of urinary biomarkers, including melatonin-sulfate, cortisol, and MT/C ratio, between case and control groups.

In addition, those urinary markers were dichotomized by medians, which were 43.23 ng/mg creatinine for melatonin, 24.06 ng/mg creatinine for cortisol, and 1.76 for MT/C ratio. Unconditional logistic regression models were used to estimate odds ratios (OR) and 95% confidence intervals (CI) for the association between the urinary markers and the risk of prostate cancer. All analyses were adjusted for age at time of questionnaire completion (<65 *vs.* ≥65 yrs), family history of PCa, personal habits (smoking, alcohol, and betel nut), and PSA level (<10 *vs.* ≥10 ng/ml). We also categorized cases into localized prostate cancer (stage T1 or T2) and advanced cancer (extra-prostatic stage T3a or higher, N1/M1)^55^, and used polytomous logistic regression models to determine the risks of developing localized and advanced prostate cancer relative to the control group^56^. Multiplicative interaction was appraised by fitting polytomous logistic models containing categorical variables, as well as their cross-products. Wald Z-tests for cross-product terms were used to evaluate the significance of multiplicative interaction for each pair of comparison. All statistical operations were performed using the SAS 9.1 statistical package; all *P*-values were two-sided and considered significant if <0.05.

## Additional Information

**How to cite this article**: Tai, S.-Y. *et al*. Urinary melatonin-sulfate/cortisol ratio and the presence of prostate cancer: A case-control study. *Sci. Rep.* **6**, 29606; doi: 10.1038/srep29606 (2016).

## Supplementary Material

Supplementary Information

## Figures and Tables

**Table 1:** Demographic characteristics of all participants.

N	CasesN =120	ControlsN = 240	χ^2^ test p-value
Age			0.796
<65 y/o	29 (24.2)	61 (25.4)	
≥65 y/o	91 (75.8)	179 (74.6)	
Education			0.635
high school & college	91 (75.9)	185 (78.1)	
≥university	29 (24.1)	52 (21.9)	
Marital status			0.201
married	109 (90.8)	205 (86.1)	
others	11 (9.2)	33 (13.9)	
Family history of PCa			0.352
No	98 (83.1)	203 (86.7)	
Yes	20 (16.9)	31 (13.3)	
Smoking			0.974
Never	84 (70.0)	167 (70.2)	
Former or current	36 (30.0)	71 (29.8)	
Alcohol			0.103
Never	102 (85.0)	216 (90.8)	
Former or current	18 (15.0)	22 (9.2)	
Betel nut			0.079*
Never	120 (100.0)	231 (97.5)	
Former or current	0 (0.0)	6 (2.5)	
Vitamin D supplement			0.068*
No	119 (99.2)	227 (95.4)	
Yes (>1 times/month)	1 (0.8)	11 (4.6)	
Preoperative PSA level			<0.001
<10 ng/ml	26 (21.8)	120 (52.4)	
≥10 ng/ml	93 (78.2)	109 (47.6)	

Abbreviation: PCa = prostate cancer; PSA = prostate-specific antigen.

^*^Fisher’s exact test.

**Table 2:** Association between urinary biomarkers of circadian hormone dichotomized by medians and the presence of prostate cancer.

Variables		ControlsN = 240	CasesN = 120	OR	95% CI	aOR^1^	95% CI
		**N (%)**					
Melatonin^2^							
Low		107 (44.6)	73 (60.8)	1.0	(Ref)	1.0	(Ref)
High		133 (55.4)	47 (39.2)	**0.52**	**0.33–0.81**	**0.59**	**0.35–0.99**
Cortisol^2^							
Low		130 (54.2)	50 (41.7)	1.0	(Ref)	1.0	(Ref)
High		110 (45.8)	70 (58.3)	**1.65**	**1.06–2.59**	1.43	0.90–2.28
MT/C ratio^2^							
Low		105 (43.8)	75 (62.5)	1.0	(Ref)	1.0	(Ref)
High		135 (56.2)	45 (37.5)	**0.47**	**0.30–0.73**	**0.46**	**0.27–0.77**
MT/C ratio^2^	PSA level						
High	<10	64 (27.9)	10 (8.4)	1.0	(Ref)	1.0	(Ref)
Low	<10	56 (24.5)	16 (13.4)	1.82	0.77–4.35	1.92	0.79–4.64
High	≥10	62 (27.1)	34 (28.6)	**3.51**	**1.60–7.71**	**3.61**	**1.62–8.07**
Low	≥10	47 (20.5)	59 (49.6)	**8.03**	**3.72–17.33**	**8.82**	**3.98–19.55**

Abbreviation: MT/C ratio = melatonion/cortisol ratio.

^1^Adjusting for age (<65 *vs.* ≥65 yr), personal habits of smoking, alcohol, betel nut, family history of prostate cancer, and prostate-specific antigen level (<10 *vs.* ≥10 ng/ml).

^2^Medians of 43.23 ng/mg creatinine for melatonin, 24.06 ng/mg creatinine for cortisol, and 1.76 for MT/C ratio.

**Table 3:** Association between urinary biomarkers of circadian hormone dichotomized by medians and clinical staging of prostate cancer.

Factor/category		ControlN = 240	Localized^1^N = 51	Advanced^1^N = 69	Localized^1^ *vs.* control		Advanced^1^ *vs.* control		Advanced^1^ *vs.* localized^1^	
		N (%)			aOR^2^	(95% CI)	aOR^2^	(95% CI)	aOR^2^	(95% CI)
Melatonin^3^										
Low		107 (44.6)	27 (52.9)	46 (66.7)	1.0	(Ref)	1.0	(Ref)	1.0	(Ref)
High		133 (55.4)	24 (47.1	23 (33.3)	0.72	0.38–1.36	**0.49**	**0.26**–**0.89**	0.59	0.27–1.28
Cortisol^3^										
Low		130 (54.2)	24 (47.1)	26 (37.7)	1.0	(Ref)	1.0	(Ref)	1.0	(Ref)
High		110 (45.8)	27 (52.9)	43 (62.3)	1.28	0.68–2.41	**1.96**	**1.07**–**3.57**	1.87	0.87–4.01
MT/C ratio^3^										
Low		105 (43.8)	27 (52.9)	48 (69.6)	1.0	(Ref)	1.0	(Ref)	1.0	(Ref)
High		135 (56.2)	24 (47.1)	21 (30.4)	0.63	0.33–1.20	**0.33**	**0.17**–**0.62**	**0.44**	**0.20**–**0.99**
Preoperative PSA										
<10 ng/ml		120 (52.4)	18 (36.0)	8 (11.6)	1.0	(Ref)	1.0	(Ref)	1.0	(Ref)
≥10 ng/ml		109 (47.6)	32 (64.0)	61 (88.4)	**2.13**	**1.10**–**4.15**	**8.17**	**3.77**–**17.72**	**3.88**	**1.49**–**10.09**
MT/C ratio^3^	PSA level									
High	<10	64 (27.9)	8 (16.0)	2 (2.9)	1.0	(Ref)	1.0	(Ref)	1.0	(Ref)
Low	<10	56 (24.5)	10 (20.0)	6 (8.7)	1.44	0.52–3.97	3.71	0.71–19.44	2.57	0.40–16.64
High	≥10	62 (27.1)	15 (30.0)	19 (27.5)	2.16	0.85–5.46	**9.36**	**2.04**–**42.91**	4.34	0.79–23.93
Low	≥10	47 (20.5)	17 (34.0)	42 (60.9)	**3.41**	**1.34**–**8.69**	**32.06**	**7.17**–**143.29**	**9.41**	**1.77**–**50.07**
*P* for multiplicative interaction					0.888		0.930		0.871	

Abbreviation: MT/C ratio = melatonion/cortisol ratio; PSA = prostate-specific antigen.

^1^Localized PCa defined as stage T1 or T2 at diagnosis; Advanced PCa defined as stage T3 or T4 and or LN or distant metastases at diagnosis.

^2^Adjusting for the same variables in Table 2.

^3^Medians of 43.23 ng/mg creatinine for melatonin, 24.06 ng/mg creatinine for cortisol, and 1.76 for MT/C ratio.
