# Supplementary material for: Urinary melatonin-sulfate/cortisol ratio and the presence of prostate cancer: A case-control study
Source: Sci Rep. 2016 Jul 8;6:29606. doi: 10.1038/srep29606 (PMC4937372; doi:10.1038/srep29606)
Supplement: Supplementary Information [file srep29606-s1.pdf]

Supplementary Material

Urinary melatonin-sulfate / cortisol ratio and the presence of prostate cancer: A case-control study

Shu-Yu Tai[1,2,3,4] , Shu-Pin Huang[5,6,7], Bo-Ying Bao[8,9,10] & Ming-Tsang Wu[4,11,12]

[1]Graduate Institute of Medicine, College of Medicine, Kaohsiung Medical University, Kaohsiung, Taiwan. [2]Department of Family Medicine, School of Medicine, College of Medicine, Kaohsiung Medical University, Kaohsiung, Taiwan. [3]Department of Family Medicine, Kaohsiung Municipal Ta-Tung Hospital, Kaohsiung, Taiwan. [4]Department of Family Medicine, Kaohsiung Medical University Hospital, Kaohsiung Medical University, Kaohsiung, Taiwan. [5]Department of Urology, Kaohsiung Medical University Hospital, Kaohsiung Medical University, Kaohsiung, Taiwan. [6]Department of Urology, Faculty of Medicine, College of Medicine, Kaohsiung Medical University, Kaohsiung, Taiwan. [7]Institute of Biomedical Science, National Sun Yat-Sen University. [8]Department of Pharmacy, China Medical University, Taichung, Taiwan. [9]Sex Hormone Research Center, China Medical University Hospital, Taichung, Taiwan. [10]Department of Nursing, Asia University, Taichung, Taiwan. [11]Department of Public Health, Kaohsiung Medical University, Kaohsiung, Taiwan. [12]Center of Environmental and Occupational Medicine, Kaohsiung Municipal Hsiao-Kang Hospital, Kaohsiung

Medical University, Kaohsiung, Taiwan.

Running title: Melatonin and the Prostate Cancer

Correspondence and requests for materials should be addressed to M.T.W. Room 721, CS Building, 100 Shih-Chuan 1st Road, Kaohsiung, Taiwan. Tel: +886-7-312-1101 ext. 2141 ext. 55; fax: +886-7-3221806 (email: 960021@ms.kmuh.org.tw or e_encourage@yahoo.com)

Supplementary Table S1. Joint risks of urinary melatonin or cortisol level and the PSA level associated with the presence of prostate cancer.

**Factor/category**		**Controls N=240**	**Cases N=120**				
		**N (%)**		**OR**	**95% CI**	**aOR[1]**	**95% CI**
**Melatonin[2]**	**PSA level**						
High	< 10	70 (30.6)	12 (10.1)	1.0	(Ref)	1.0	(Ref)
Low	< 10	50 (21.8)	14 (11.7)	1.63	0.70-3.83	1.63	0.69-3.88
High	≥ 10	54 (23.6)	34 (28.6)	**3.67**	**1.74-7.76**	**3.71**	**1.73-7.94**
Low	≥ 10	55 (24.0)	59 (49.6)	**6.26**	**3.06-12.78**	**6.58**	**3.16-13.71**
**Cortisol[2]**	**PSA level**						

Low	< 10	61 (26.6)	11 (9.2)	1.0	(Ref)	1.0	(Ref)
High	< 10	59 (25.8)	15 (12.6)	1.41	0.60-3.32	1.42	0.60-3.39
Low	≥ 10	62 (27.1)	39 (32.8)	**3.49**	**1.64-7.43**	**3.71**	**1.71-8.04**
High	≥ 10	47 (20.5)	54 (45.4)	**6.37**	**3.00-13.51**	**6.33**	**2.94-13.61**

[1]Adjusting for age (< 65 *vs.* ≥ 65 yr), personal habits of smoking, alcohol, betel nut, family history of prostate cancer, and prostate-specific antigen level (<10 *vs.* ≥ 10 ng/ml).

[2]Medians of 43.23 ng/mg creatinine for melatonin, 24.06 ng/mg creatinine for cortisol, and 1.76 for MT/C ratio.

Supplementary Table S2. Joint risks of urinary melatonin or cortisol level and the PSA level associated with clinical staging of prostate cancer.

**Factor/category**		**Control N=240**	**Localized[1] N=51**	**Advanced[1] N=69**	**Localized[1] vs. control**		**Advanced[1] vs. control**		**Advanced[1] vs. Localized[1]**	
			N (%)		aOR[2]	(95%CI)	aOR[2]	(95%CI)	aOR[2]	(95%CI)
**Melatonin**[3]	**PSA level**									
High	< 10	70 (30.6)	9(18.0)	3 (2.5)	1.0	(Ref)	1.0	(Ref)	1.0	(Ref)
Low	< 10	50 (21.8)	9 (18.0)	5 (4.2)	1.38	0.51-3.77	2.34	0.53-10.35	1.69	0.30-9.42
High	≥ 10	54 (23.6)	14 (28.0)	20 (16.8)	2.24	0.91-5.55	**8.11**	**2.25-29.33**	3.62	0.82-15.95
Low	≥ 10	55 (24.0)	18 (36.0)	41 (34.5)	**2.92**	**1.21-7.04**	**18.06**	**5.19-62.85**	**6.19**	**1.47-25.97**

	**PSA**									
**Cortisol** [3]	**level**									
Low	< 10	61 (26.6)	7 (14.0)	4 (5.8)	1.0	(Ref)	1.0	(Ref)	1.0	(Ref)
High	< 10	59 (25.8)	11 (22.0)	4 (5.8)	1.61	0.58-4.48	1.05	0.25-4.47	0.66	0.12-3.57
Low	≥ 10	62 (27.1)	17 (34.0)	22 (31.9)	**2.87**	**1.11-7.42**	**5.11**	**1.61-16.22**	1.78	0.44-7.22
High	≥ 10	47 (20.5)	15 (30.0)	39 (56.5)	**2.91**	**1.09-7.75**	**12.74**	**4.17-38.91**	**4.38**	**1.10-17.36**

PSA = prostate-specific antigen.

[1]Localized PCa defined as stage T1 or T2 at diagnosis; Advanced PCa defined as stage T3 or T4 and or LN or distant metastases at diagnosis

[2]Adjusting for the same variables in Table 2.

[3]Medians of 43.23 ng/mg creatinine for melatonin, 24.06 ng/mg creatinine for cortisol, and 1.76 for MT/C ratio.
